# Non-Coding RNAs in Lung Tumor Initiation and Progression

**DOI:** 10.3390/ijms21082774

**Published:** 2020-04-16

**Authors:** Ruben Mercado Santos, Cerena Moreno, Wen Cai Zhang

**Affiliations:** Department of Cancer Division, Burnett School of Biomedical Sciences, College of Medicine, University of Central Florida, 6900 Lake Nona Blvd, Orlando, FL 32827, USA; ruben.mercado30@Knights.ucf.edu (R.M.S.); cerenamoreno@Knights.ucf.edu (C.M.)

**Keywords:** microRNA, long non-coding RNA, lung cancer, immortalization, tumor initiation, tumor progression, cancer metabolism, oncogene, RNA editing, RNA modifications

## Abstract

Lung cancer is one of the deadliest forms of cancer affecting society today. Non-coding RNAs, such as microRNAs (miRNAs), long non-coding RNAs (lncRNAs), and circular RNAs (circRNAs), through the transcriptional, post-transcriptional, and epigenetic changes they impose, have been found to be dysregulated to affect lung cancer tumorigenesis and metastasis. This review will briefly summarize hallmarks involved in lung cancer initiation and progression. For initiation, these hallmarks include tumor initiating cells, immortalization, activation of oncogenes and inactivation of tumor suppressors. Hallmarks involved in lung cancer progression include metastasis and drug tolerance and resistance. The targeting of these hallmarks with non-coding RNAs can affect vital metabolic and cell signaling pathways, which as a result can potentially have a role in cancerous and pathological processes. By further understanding non-coding RNAs, researchers can work towards diagnoses and treatments to improve early detection and clinical response.

## 1. Introduction 

Lung cancer continues to be one of the leading causes of cancer death worldwide [1,2]. In the United States alone, it is estimated that 228,820 people will be diagnosed with lung cancer in the year 2020 and that 135,720 individuals will die from lung cancer [2,3]. Although the average 5-year survival rate of patients with lung cancer has increased from 14% (1989–1995) [4] to 19% (2009–2015) [2] among patients in the United States during last two decades, further research is being conducted to increase the long-term survival of these patients. Non-coding RNAs have become an area of interest, as their expression is altered in specific cancers, hence implicating them as possible controllers of tumor initiation [5] and progression [6]. This review will briefly summarize hallmarks involved in lung tumorigenesis and metastasis and then specifically focus on microRNAs (miRNAs), long non-coding RNAs (lncRNAs), and circular RNAs (circRNAs) that have been found to be dysregulated in lung tumors. This can occur through different mechanisms of action in affecting the aforementioned hallmarks. Furthermore, technologies currently being developed to further strengthen our understanding of non-coding RNAs will be discussed. At last, the therapeutic value of targeting these non-coding RNAs will be evaluated. 

## 2. Lung Cancer

Lung cancer is divided into two main categories: Non-small cell lung carcinoma (NSCLC), which currently accounts for 85% of new lung cancer diagnoses and small-cell lung carcinoma (SCLC), which accounts for 15% of lung cancer diagnoses [7]. NSCLC is further divided into three different forms, including adenocarcinoma, squamous-cell carcinoma, and large-cell carcinoma [7]_._ The reasoning behind these divides is due to histopathological and clinical differences [8]. For instance, in regard to genetic mutations, the main causes of continued proliferation of adenocarcinoma, squamous-cell carcinoma, and SCLC differ. This is shown by the observance that adenocarcinoma is commonly caused by *liver kinase B1* (STK11) mutations, *epidermal growth factor receptor* (EGFR) kinase domain mutations, *tyrosine-protein kinase MET* (MET) amplification, *Kirsten rat sarcoma viral* (KRAS) mutations, and *anaplastic lymphoma kinase* (ALK) mutations. Alternatively, squamous-cell carcinoma is commonly caused by *EGFR* amplification, *phosphatidylinositol-4,5-bisphosphate 3-kinase catalytic subunit alpha* (PIK3CA) amplification and *MET* amplification [7]. In addition, SCLC is commonly caused by *MET* mutations and *PIK3CA* amplification [7]. Yet, other abnormalities such as *tumor protein p53* (TP53) mutations are highly found throughout all the aforementioned types of lung cancers [9]. Other characteristics shared by the different types and subtypes of lung cancer are the different factors linked to their onset such as non-genetic abnormalities including smoking behaviors, exposure to radon gas, asbestos, radiation, air pollution and diesel exhaust [8] along with individual-based factors such as aging, obesity, lack of physical activity and reproductive changes [1,10]. Patients with extensive-stage SCLC typically undergo immunotherapy in combination with chemotherapy [11,12], while patients with NSCLC typically receive treatment options such as chemotherapy, immunotherapy, and targeted therapy drugs such as EGFR and anaplastic lymphoma kinase (ALK) inhibitors [13]. Different from other receptor tyrosine kinases such as EGFR and ALK, it has been challenging to target KRAS directly due to a high affinity of KRAS protein for guanosine triphosphate (GTP)/guanosine diphosphate (GDP) and the lack of a clear binding pocket [14]. Recently, small molecular inhibitors against *KRAS Gly12Cys (G12C)* have been developed [15] and showed promises in human clinical trials, including AMG510 [16,17] and MRTX849 [18,19]. These inhibitors selectively modify the mutant cysteine residue in GDP-bound KRAS G12C and inhibit GTP-loading and downstream KRAS-dependent signaling [20]. In phase I clinical trial with AMG510, the therapy is promising with a partial response [21] in two patients and a stable disease in other two patients [16]. Thus, genetic mutations/signaling pathways-based targeted therapies for lung cancer will demonstrate promise of success in the future. 

## 3. Lung Tumor Initiation

Tumor-initiating cells (TICs), or cancer stem cells (CSCs), have unique characteristics such as the ability to self-renew, give rise to alternative progeny, initiate and maintain tumors, and activate anti-apoptotic and pro-immortalization pathways [22]. The majority of these characteristics are also seen in stem cells [22]. It is due to this similarity that there are a couple ways implemented to identify TICs such as marker-based strategy by isolating cells with similar cell surface markers seen in normal stem cells as well as marker independent strategy to identify the side populations [23]. The reason underlying the creation of different models and assays to determine TICs is due to their roles in tumor initiation and drug resistance. TICs are able to initiate tumorigenesis by regulating self-renewal genes that can lead to uncontrolled growth. For example, through the sphere formation model, CD44^+^ cells in NSCLC were found to initiate tumorigenesis by aberrant expression of octamer binding transcription factor 4 (OCT4), SRY-box transcription factor 2 (SOX2), and Nanog homeobox (NANOG), genes known to be regulators of self-renewing and differentiation abilities in cells [24]. Other currently known biomarkers of lung cancer TICs include CD133^+^ [25], CD166^+^ [26], and CD24^+^ITGB4^+^Notch^hi^ [27]. Furthermore, signaling pathways that act as either oncogenes or tumor suppressors in lung cancer, such as notch, wingless-related integration site and hedgehog have been found to be abnormally expressed in TICs, indicating TICs expression of these signaling pathways can lead to tumorigenesis in lung cancer [28]. TICs can become drug resistant by going into a quiescent state (side population) that allows them to not be targeted by chemotherapeutic agents that target actively dividing cells [29]. One of the factors that allows side populations to enter a non-dividing stage is epithelial–mesenchymal transition (EMT) [30]. CD44^+^CD90^+^ side populations in NSCLC and SCLC have been shown to increase the expression of the mesenchymal markers N-Cadherin and Vimentin, which led to promotion of EMT and hence drug resistance in these cell lines [24]. CD133^+^ cells in NSCLC have been shown to express high levels of ATP-binding cassette G2 [16], a transporter that can lower intercellular drug concentration through efflux of drugs [24,31]. Other studies have shown CD133^+^ of being capable of self-renewal, hence implicating CD133^+^ in drug resistance and the ability to recreate original tumor growth [32]. An overall problem in targeting TICs is that their microenvironment induces changes to the phenotype of TICs. This plasticity means that eradication of TICs may lead to the creation of TICs from dormant ones, and is why Plaks and colleagues advocate the targeting of TICs microenvironment, which includes some of the aforementioned pathways and genes [33].

Overall, the first step in lung tumor initiation is for a cell to become immortalized [34], which occurs by ensuring its telomeric DNA is not shortened through the action of telomerase [35]. Historically, it was believed that the upregulation/activation of the gene *telomerase reverse transcriptase* (hTERT) solely leads to immortalization [35], but recent research suggests that immortalization is a two-step process in which hTERT promoter mutations and further subsequent upregulation of telomerase occurs [36,37]. For instance, tracheobronchial epithelial cells were shown to not become immortalized by the expression of hTERT alone, exemplifying other factors are involved in lung epithelial cell immortalization [38]. Others suggest that cyclin dependent kinase 4 (CDK4) is also involved in immortalization as co-transfection of CDK4 and hTERT were needed in order for human bronchial epithelial cells to become immortal [34]. After cells become immortalized, genetic mutations of oncogenes and the inactivation of tumor suppressors are the next critical step in lung tumor initiation for both NSCLC and SCLC [39]. The alteration of certain oncogenes and the inactivation of certain tumor suppressor cells tends to be more prevalent depending on the type and subtype of lung cancer. For instance, adenocarcinoma is generally induced in part to alterations in *KRAS*, *ALK*, *ROS proto-oncogene 1* (ROS1), *Ret proto-oncogene* (RET), *neurotrophic receptor kinase 1* and *neuregulin* [40]. Furthermore, squamous cell carcinoma is commonly a byproduct of mutations in *TP53, cyclin dependent kinase inhibitor 2A (CDKN2A)*, *SOX2*, and *akt serine/thereonine kinase* (*AKT*) [41]. In SCLC, mutations of *rb transcriptional corepressor 1* (*RB1*) and *TP53* [42] are a more common occurrence compared to NSCLC [43]. *TP53* is a well-known tumor suppressor, whose alteration leads to uncontrolled cellular growth [44]. Conversely, *EGFR* is an oncogene that has been widely examined and whose activation, regardless of the pathway/mechanism, is a main culprit in lung tumor initiation, evidenced by its mutation being observable in 43%-89% of cases regarding NSCLC [45,46]. Overall, lung tumor initiation involves immortalization followed by transformation mediated by activation of oncogenes and inactivation of tumor suppressors.

## 4. Lung Tumor Progression

Metastasis initiating cells (MICs) are TICs with the ability to initiate a secondary tumor growth site [47]. For example, breast cancer stem cells with the cell markers CD44^+^ and CD24^−/low^ have been shown to initiate tumorigenesis after chemotherapy and begin the process of metastasizing to the lung [48]. Yet, Celia-Terrassa et al. contend that if TICs are to be involved in both initiation and metastasis, they must have additional capabilities seen in TICs in order to survive conditions faced when they escape the primary tumor site [49]. For instance, in addition to the self-renewal and plasticity capabilities described before with TICs, researchers theorize that MICs must have mesenchymal-to-epithelial capabilities in order to attach at a secondary metastatic lesion [49]. Brain metastasis initiating cells from the lung increase epithelial development to colonize the brain through the targeting of genes such as forkhead box C2, noggin and fibroblast growth factor receptor 2 [50]. In addition, Celia-Terrassa and colleagues also contend that MICs must be able to reprogram their metabolism as primary and secondary tumor sites that can have different metabolites available [49]. This idea of reprogramming to combat stress in the cellular environment is a common occurrence in lung cancer metabolism via aerobic glycolysis and oxidative phosphorylation, so with an abundance of glucose present in lung cancer the MICs are able to have enough resources to further progress [51].

After a cell becomes immortalized and has become transformed through the activation of oncogenes and inactivation of tumor suppressor genes, research suggest the first step in metastasis begins with tumor cells invading the stroma of its primary tumor site [52]. After successful invasion of the stroma, a tumor cell must form new blood vessels before it spreads to other areas of the body by releasing protein factors that are members of the vascular endothelial growth factor (VEGF) family and binding to VEGF receptors [53]. Although primary tumors promote angiogenesis, they still tend to exceed their blood supply and subsequently hypoxic conditions are induced [54]. Typically, cells will undergo apoptosis under such conditions, but tumor cells are able to upregulate both hypoxia-inducible factor 1 alpha (HIF-1*α*) and hypoxia-inducible factor 2 alpha (HIF-2*α*), which synergistically leads to the transcription of VEGF and hence the creation of more blood vessels [55]. Specific types of cancer cells are also able to activate other pathways in order to survive the hypoxic conditions they create. For instance, upregulated expressions of Notch 1 in hypoxic lung adenocarcinoma cells activate insulin-like growth factor 1 receptor (IGF1R) via binding to the IGF1R promoter directly leading to activation of Akt1, which allows the tumor cells to survive under hypoxic parameters [56]. It is important to note that angiogenesis and hypoxia can lead to EMT, which in addition to affecting TICs (initiation) can also give tumor cells properties of invasion and movement that aid in metastasis [57]. The next step before a tumor cell can migrate and expand in secondary areas is for these cells to evade their hosts’ immune response, usually done by tumor cells through the evasion of cytotoxic T lymphocytes (CTLs), antigen presenting cells (APCs) and other components of the innate immune response [52,56]. The ability of a tumor cell to undergo the aforementioned changes allows it to progress to the point where it is ready to migrate to other areas where it can begin to metastasize. Once the tumor cell is ready to leave the blood supply, it exits through a process called extravasation, where it subsequently creates new blood vessels and begins to metastasize at the secondary tumor location [58].

Most of the therapeutic approaches against lung cancer include the targeting of processes that allow tumors to initiate and progress. The main therapies include EGFR tyrosine kinase inhibitors (TKIs) (gefitinib [59,60], afitinib [61], and osimertinib [61]), ALK inhibitors (crizotinib [62], certinib [62], alectinib [62], and lortatinib [63]), and KRAS inhibitors (AMG510 [16], MRTX849 [18] and BI-2852 [64]). Yet, many tumor cells develop resistance. Cells resistant to EGFR TKIs may undergo T790M substitution [65] and C797S mutation [66,67]. A mutation that aids EGFR resistance against all generations of EGFR-TKIs is tyrosine-protein kinase Met (c-MET) gene amplification [68]. Since both of these mutations work hand in hand, previous and recent research suggests co-targeting both EGFR and c-MET in lung cancer [69]. Another common mutation is through the *Echinoderm microtubule-associated protein-like 4* (EML4) and *ALK* mutations [70], but resistance prevailed due to mutations in C1156Y and L1196M within EML4-ALK [70]. KRAS in NSCLC include G12C (most common), G12D, G13D and G12V [71]. Currently resistance against AMG510 is due in part to the fact that G12C inhibitor only binds to the inactive state of G12C. After G12C inhibitor is introduced into a culture they induce lower overall KRAS activity, but after a brief period KRAS activity resumes due to the production of new G12C that is in an active state, making G12C inhibitor obsolete, as they are not able to bind to their targets and inhibit them [72].

In addition to drug resistance via genetic mutations, clinicians also have to deal with drug tolerance. The reason for this concern is due to drug tolerance and drug resistance having a complex synergistic relationship in which drug-tolerant “persisters” lead to drug resistance [73]. Persisters are subpopulations of cancer cells that go into a quiescence state to avoid traditional therapeutics aimed at inhibiting tumor cell growth [74]. Recently, a study focusing on EGFR T790M^-^ drug tolerant cells found them to evolve the EGFR T790 mutation after initial survival to drugs [75]. Due to the interplay between drug tolerant and drug resistant cells, researchers have advocated targeting both of these cells to avoid persisters from also becoming resistant [75]. Researchers have begun to take this approach as after it was found that aurora kinase A, activated by TPX2 microtubule nucleation factor leads to drug tolerance of 3^rd^ generation EGFR TKIs, both EGFR TKIs and aurora kinase A inhibitors were found to induce apoptosis and lower the growth of acquired resistant cells [76]. 

## 5. Non-Coding RNA Network in Lung Cancer

miRNAs are a class of non-coding RNAs whose main function is to regulate gene expression at a post-transcriptional level [77]. miRNAs accomplish the aforementioned purpose by either binding to messenger RNA (mRNA) and disturbing mRNAs translational activity or by initiating mechanisms that promote mRNA decay factor activity that leads to increase mRNA degradation [77]. The ability of miRNAs to repress mRNA that has led to its identification as a major player in lung tumor initiation and progression [78]. Another non-coding RNA that has a key role in lung tumorigenesis and metastasis is long non-coding RNA (lncRNA). Unlike miRNAs, lncRNAs manifest themselves in lung cancer by regulating gene expression not only at the post-transcriptional level, but also at the transcriptional and epigenetic level [79]. It is important to note that lncRNAs and miRNAs are able to interact with each other, evidenced by the ability of lncRNAs to act as miRNA sponges, where lncRNAs can increase or decrease miRNAs effect on mRNA by outcompeting substrates that traditionally bind to miRNAs [80]. Circular RNA (circRNA) forms a covalently closed continuous loop and function as a sponge for a particular miRNA or RNA binding protein [81]. The roles of circRNAs in lung tumor as biomarkers and master regulators have been reviewed [82]. Several interactions between lncRNAs, circRNAs, and miRNAs will be discussed below along with non-coding RNAs effect, mediated by their upregulation or downregulation, on downstream targets implicated to have a role in lung tumor initiation and progression [80].

### 5.1. Role of Non-Coding RNAs in Lung Tumor Initiation

#### 5.1.1. Role of Non-Coding RNAs in Lung Tumor Initiating Cells

Dysregulation of certain non-coding RNAs in lung TICs control tumor initiation and progression. For instance, upregulation of miR-1246 and miR-1290 led to repression of metallothioneins, especially metallothionein 1G (MT1G), which led to increased TIC ability to initiate tumor growth and metastasis in NSCLC (Table 1) [83]. Metallothioneins play key roles in tumor growth as they regulate zinc levels required for G1/S phase transition and increase expression of matrix metalloproteinase 3 [84]. Upregulation of miR-494-3p led to activation of Notch 1 and PI3K signaling pathways, which increased TICs ability to proliferate [85]. On the other hand, miR-145 inhibits TIC proliferation by directly targeting and repressing *OCT4* mRNA [86]. Decreased levels of miR-31 and elevated levels of let-7 cooperatively inhibited growth of lung TICs through the cell cycle arrest [87]. Alternatively, signal transducer and activator of transcription 3 (STAT3) activation-induced upregulation of the lncRNA HOX transcript antisense RNA (HOTAIR) via its promoter activity promoted lung tumorigenesis through EMT (Table 2) [88]. Knocking down HOTAIR inhibits features of TICs including frequencies of side population cells and spheroid forming cells [88]. Chen and colleagues recently reviewed regulations of lncRNAs in TICs in several types of cancers [89]. Thus, it is imperative to find whether these lncRNAs are able to affect TICs involved in lung tumorigenesis to find avenues of targeting these lncRNAs. 

#### 5.1.2. Role of Non-Coding RNAs in Immortalized Genes 

Recent evidence has shown that non-coding RNAs mediate oncoprotein-induced immortalization of lung epithelial cells. Activation of Simian virus 40 small T antigen (SV40 ST) and TERT in human bronchial epithelial cells induces an upregulation of miR-27a leading to enhanced immortalization. miR-27a mediated immortalization is through repressing downstream cell cycle regulator F-box/WD repeat-containing protein 7 (FBXW7) (Table 1) [145]. On the other way, miRNAs are involved in the immortalization process through promoting expressions of immortalized gene hTERT or CDK4 directly in lung cancer. For example, downregulated miR-545 expression in NSCLC enhances expressions of CDK4 leading to enhanced cell proliferation [146]. Similarly, other tumor suppressive miRNAs including miR-486-5p [147], miR-613 [148], miR-340 [149], and miR-34b-3p [150] upregulate expression of CDK4 in NSCLC. Although few studies of non-coding RNAs involved in the immortalization process have been demonstrated in lung cancer, the aforementioned data suggests that these links could provide valuable therapeutic targets.

#### 5.1.3. Role of Non-Coding RNAs in Oncogenes and Tumor Suppressors 

The ability of non-coding RNAs to interact with oncogenes is a valuable hallmark in lung tumor initiation and progression. The first miRNA discovered was *let-7* family including *let-7a, let-7b, let-7c, let-7d, let-7e, let-7f, let-7g, let-7i*, miR-98, and miR-202 [151]. *let-7* family members bind to the 3′ untranslated regions (3′ UTR) of *RAS* oncogene and is downregulated in lung cancers (Table 1) [104]. 

Tumor suppressor control non-coding RNA expressions, and vice versa. Tumor suppressor p53 directly regulates cancer suppressive miR-34a/b/c expression via manipulating promoter activity of miR-34 in lung cancer [152]. Reduced miR-34 expression promotes lung tumor initiation via derepressing cell-cycle regulatory genes including Met and Bcl-2 [153]. On the other way, upregulation of miR-21 in NSCLC enhances KRAS-driven tumor initiation via repressing tumor suppressors including programmed cell death 4 (PDCD4) and apoptosis peptidase activating factor 1 (APAF1) [90]. Similarly, overexpression of miR-31 increases tumorigenesis via decreasing RAS p21 protein activator 1 (RASA1), sprouty related EVH1 domain containing 1/2 (SPRED1/2), and Sprouty 1/3/4 (SPRY1/3/4) in KRAS-driven NSCLC [91]. In SCLC, overexpression of the miR-17~92 cluster including seven miRNAs promotes the tumor development through repressing tumor suppressor PTEN and RB transcriptional corepressor like 2 (RBL2) [92].

Another key relationship between non-coding RNAs and lung cancer is how miRNAs can be used as diagnostic markers. The upregulation of both miR-205 and miR-375 has successfully distinguish lung squamous cell carcinoma from lung adenocarcinoma [154]. Further research on miRNAs and lncRNAs as biomarkers in histological subtyping is advocated as more patient-specific treatments can be provided once the tumor type is known. 

#### 5.1.4. Role of Non-Coding RNAs in RNA Editing 

**Figure 1:**
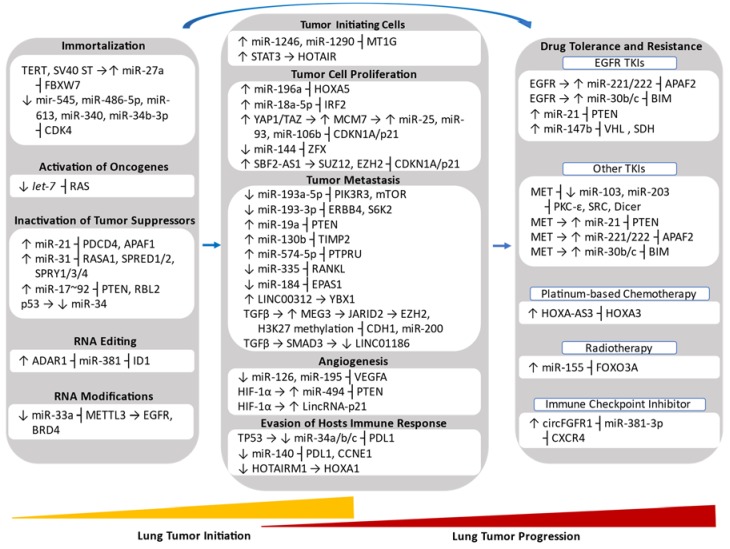
Non-coding RNAs involved in lung cancer initiation and progression along with the hallmark they affect. ↑, upregulation; ↓, downregulation; →, promotion; ┤, inhibition. Abbreviations: Apoptotic peptidase activating factor 1/2 (APAF1/2), BCL-2-like protein 11 (BIM), bromodomain-containing protein 4 (BRD4), cyclin E1 (CCNE1), E-cadherin (CDH1), cyclin dependent kinase 4 (CDK4), cyclin dependent kinase inhibitor 1A (CDKN1A), C-X-C motif chemokine receptor 4 (CXCR4), endoribonuclease Dicer (DICER), epidermal growth factor receptor (EGFR), endothelial PAS domain protein 1 (EPAS1), Erb-B2 receptor tyrosine kinase 4 (ERBB4), enhance of zeste 2 polycomb repressive complex 2 subunit (EZH2), F-box and WD repeat domain containing 7 (FBXW7), forkhead box O3 (FOXO3A), hypoxia inducible factor 1 alpha (HIF-1α), homeobox A3 (HOXA3), homeobox A5 (HOXA5), inhibitor of DNA binding 1 (ID1), interferon regulatory factor 2 (IRF2), Jumonji and AT-rich interaction (JARID2), minichromosome maintenance complex component 7 (MCM7), methyltransferase like 3 (METTL3), SMAD Family Member 3 (SMAD3), mechanistic target of rapamycin kinase (mTOR), metallothionein 1G (MT1G), protein tyrosine phosphatase receptor type U (PTPRU), programmed cell death 1 ligand 1 (PD-L1), programmed cell death 4 (PDCD4), phosphatidylinositol-3-kinase regulatory subunit 3 (PIK3R3), phosphatase and tensin homolog (PTEN), protein kinase C epsilon (PKC-ε), tumor necrosis factor ligand superfamily member 11 (RANKL), Ras p21 protein activator (RASA1), retinoblastoma-like protein 2 (RBL2), succinate dehydrogenase (SDH), sprouty related EVH1 domain containing 1/2 (SPRED1/2), sprouty 1 (SPRY1/3/4), SRC proto-oncogene non-receptor tyrosine kinase (SRC), signal transducer and activator of transcription 3 (STAT3), SUZ12 polycomb repressive complex 2 subunit (SUZ12), Simian virus 40 small T antigen (SV40 ST), transforming growth factor beta 1 (TGFβ), tissue inhibitor of metalloproteinases 2 (TIMP2), tumor protein 21 (TP21), tumor protein 53 (TP53), telomerase reverse transcriptase (TERT), vascular endothelial growth factor A (VEGFA), von Hipper-Lindau tumor suppressor (VHL), yes associated protein 1/tafazzin (YAP1/TAZ), Y-Box binding protein 1 (YBX1), zinc finger protein X-linked (ZFX).

RNA editing is the process of an RNA editor changing nucleotide sequences in RNA to change the way a codon is read by ribosomes [155]. In addition to regulating mRNA, RNA editors can also affect non-coding RNAs such as miRNAs and lncRNAs expression, and hence play a role in cancer initiation and progression [156]. The commonly known RNA editing event is adenosine to inosine (A-to-I) conversion catalyzed by adenosine deaminase acting on RNA (ADAR) (Figure 1) [157]. Previous studies have demonstrated that ADAR1 inhibits miRNA processing globally through forming ADAR1/Dicer complex via the RNA interference machinery [158]. Although most ADAR editing occurs in non-coding regions in lung cancer, few studies have shown the crosstalk between ADAR and non-coding RNAs [159]. Anadon et al. have demonstrated that *ADAR1* gene amplification in NSCLC promotes tumor growth via enhanced A-to-I editing of RNA transcripts including miR-381 [160]. It is consistent to previous finding that reduced miR-381 expression promotes tumor cells proliferation and resistance to platinum-based chemotherapy via upregulating inhibitor of DNA binding 1 (ID1) levels directly in NSCLC [161]. Furthermore, Nigita et al. have shown dysregulated miRNA editing events in both tumor tissues and circulating exosomes in NSCLC, such as miR-411-5p [162]. As for ADAR editing on lncRNAs, a database of “LNCediting” has been developed to identify lncRNAs that undergo ADAR editing [163]. LNCediting could potentially predict roles of interactions of ADAR and lncRNAs in lung tumor initiation and progression. A role in RNA editing of non-coding RNAs has been established and found to have wide-ranging effects on prognosis. An avenue in which regulation of RNA editing to hence modify non-coding RNAs should be explored to inhibit negative effects of RNA editing and increase patient prognosis.

#### 5.1.5. Role of Non-Coding RNAs in RNA Modifications

RNA modifications, including modification of the 5′ cap and 3′ end of mRNA along with their biological significance have been well studied [164]. Recently, N^6^-methyladenine (m^6^A) modifications affecting internal modification of mRNAs have been gaining more attention due to their ability to affect cancer [164]. For example, m^6^A inducers such as methyltransferase-like 3 (METTL3) have been shown to act as an oncogene by increasing the translation of EGFR in NSCLC (Figure 1) [165]. It has been identified that m^6^A modifications regulate miRNA biogenesis, and vice versa. For instance, primary microRNAs (pri-miRNAs) are processed into miRNAs by both DiGeorge syndrome critical region gene 8 microprocessor complex subunit (DGCR8) and drosha ribonuclease type II (DROSHA) [166]. METTL3 increases methylation in pri-miRNAs allowing for DGCR8 to bind to and promotes pri-miRNAs processing [166]. Non-coding RNAs also regulate these m^6^A modifications in lung cancer. Downregulated miR-33a binds to the 3′ UTR of *METTL3* and increases METTL3 expression levels [105]. The decrease in levels of METTL3 induced apoptosis and slowed tumorigenesis in NSCLC [105] due that METTL3 promotes translation of oncogenic mRNAs including EGFR [165] and bromodomain-containing protein 4 (BRD4) [167] (Figure 1). It is of consequence to also note that m^6^A can affect ADAR editing by m^6^A suppression, which has been correlated to induce increased ADAR editing, this insight is noteworthy as it can be correlated that m^6^A downregulation can lead to the aforementioned effects of ADAR editing on cancers [168]. With a better understanding of the interactions between RNA modification and their effect on non-coding RNAs, they could serve as potential targets to inhibit lung tumor initiation and progression. 

### 5.2. Role of Non-Coding RNAs in Lung Tumor Progression 

#### 5.2.1. Role of Non-Coding RNAs in Tumor Cell Proliferation

miR-196a was shown to act as an oncogenic miRNA as its upregulation in NSCLC led to inhibition of homeobox A5, which led to proliferation of NSCLC cells (Figure 1; Table 1) [93]. Upregulated miR-18a expression decreases interferon regulatory factor 2 (IRF2) activity leading to decreased cell apoptosis and enhanced cell proliferation in NSCLC tumor cells [94]. Upregulated expression of lncRNA SBF2-AS1 promotes NSCLC cell proliferation via direct binding to SUZ12 polycomb repressive complex 2 subunit (SUZ12) and enhancer of zeste 2 polycomb repressive complex 2 subunit (EZH2) using an RNA immunoprecipitation approach (Figure 1; Table 2) [112]. The enrichment of both SUZ12 and EZH2 reduces protein levels of cyclin dependent kinase inhibitor 1A (CDKN1A)/p21 linking DNA damage to cell cycle arrest [169]. Similarly, upregulation of *MCM7* gene and its hosted miR-25, miR-93, and miR-106b cluster via binding to minichromosome maintenance complex component 7 (MCM7) promoter by activated yes associated protein 1/tafazzin (YAP1/TAZ) led to increased NSCLC tumor cell proliferation through inhibiting p21 directly [95]. In addition, reduced expression of miR-144 in NSCLC promote tumor cells proliferation and inhibits apoptosis via upregulating zinc finger X-chromosomal protein (ZFX) [170] (Figure 1). It suggests that non-coding RNAs play important roles in lung tumor cell proliferation.

#### 5.2.2. Role of Non-Coding RNA in Tumor Metastasis

Pertaining to metastasis, research shows that suppression of the tumor suppressor gene, epithelial cadherin (CDH1), is a key factor in EMT as it allows for the tumor cell to detach from its primary tumor site and metastasize in a secondary site [171]. As illustrated before, MICs have the ability to undergo mesenchymal–epithelial transition to colonize a secondary site and can subsequently target mechanisms to proliferate [50]. One of these mechanisms includes increased expression of signal transducers and activators of transcription 3, which can bind to and increase miR-21 levels, which in turn allows MICs to proliferate and metastasize [172]. In NSCLC, decreased miR-193a-5p and miR-193a-3p expressions linked to enhanced tumor metastasis through upregulating PIK3R3 and mTOR as well as ERBB4 and S6K2, respectively (Figure 1; Table 1) [106]. Alternatively, upregulated miR-19a expression in NSCLC promotes tumor cell metastasis via repressing PTEN [96]. Further study showed that PTEN inactivation led to nuclear translocation of β-catenin and Snail/Slug in lung cancer cells [173]. The following miRNAs have been found to have a role in tumor cells ability to proliferate/invade/migrate, but the exact hallmark and mechanism is not yet known. miR-130b also acts as an oncogenic miRNA as its upregulation leads to the decreased expression of tissue inhibitor of metalloproteinase-2 (TIMP-2), which leads to increased activity in matrix metallopeptidase 2 and enhanced tumor metastasis [97]. As for SCLC, miR-355 downregulation directly induces increased expression of tumor necrosis factor ligand superfamily member 11 (RANKL) leading to bone metastasis [107]. miR-574-5p is an oncogenic miRNA in SCLC as its promotion leads to repression of protein tyrosine phosphatase receptor type U (PTPRU), which in turn increases tyrosine phosphorylation of β-catenin [98]. In contrast, miR-184 has demonstrated downregulation in SCLC and acts as a tumor suppressive miRNA as it represses endothelial PAS domain protein 1 (EPAS1)/HIF-2α leading to β-catenin activation [98]. Activation of β-catenin further regulate E-cadherin pathway linking to tumor cell metastasis [174]. In addition, highly invasive tumor cells promote metastasis by forming fluid-conducting channels, termed as vasculogenic mimicry. For instance, upregulated LINC00312 expression in lung adenocarcinoma tissue induces tumor metastasis and vasculogenic mimicry through direct binding to Y-Box binding protein 1 (YBX1) (Figure 1; Table 2) [113]. Elevated JARID2 recruits EZH2 and induces H3K27 methylation, which repress CDH1 and miR-200 family [114]. In contrast, TGFβ-induced SMAD family member 3 (SMAD3) activation repress expression of lncRNA LINC01186 in NSCLC cells leading to EMT and metastasis [123]. As shown, EMT, tumor cell proliferation, and invasion are key hallmarks involved in lung cancer metastasis that can be promoted by non-coding RNAs. Further research is advocated to block non-coding RNAs ability to spread cancers towards secondary sites through these specific hallmarks [175]. 

#### 5.2.3. Role of Non-Coding RNA in Angiogenesis

As previously mentioned, VEGF has a critical role in angiogenesis. miR-126 and miR-195 are both downregulated in NSCLC which lead to angiogenesis as both miR-126 [108] and miR-195 [109] can bind to the 3′ UTR of VEGFA and inhibit its overexpression (Figure 1; Table 1). Tumor cells have the ability to promote angiogenesis during hypoxic conditions. Mao et al. reported that in NSCLC hypoxic conditions induce upregulation of miR-494 in tumor cells that transmit miR-494 into vascular endothelial cells via a microvesicle-mediated route. Repression of PTEN in endothelial cells by miR-494 led to increased phosphorylation of AKT serine/threonine kinase and hence activated the AKT serine/threonine kinase/endothelial nitric oxide synthase pathway that led to an increase in pro-angiogenic factors such as VEGF [99]. lncRNAs are also able to upregulate VEGF expression levels. In lung adenocarcinoma, HIF-1α activation-induced lincRNA-p21 upregulation promotes angiogenesis through increased production of VEGFA, matrix metallopeptidase 2, and fibroblast growth factor (Figure 1; Table 2) [125]. This insight is related to a previously examined theme that angiogenesis is related to other hallmarks of lung cancer such as hypoxia and EMT, and this suggests that there is high duality of non-coding RNAs that can target different hallmarks in order to advance or suppress oncogenic activity.

#### 5.2.4. Role of Non-Coding RNA in Evasion of Host Immune System

Immune checkpoints, or regulatory signals, are important for the regulation of T-cell response. The most extensively studied for targeted immunotherapy in lung cancer include inhibitory receptors cytotoxic T-lymphocyte-associate protein 4 (CTLA4), programmed death protein 1 (PD1), and programmed death ligand 1 (PD-L1) [176]. Additionally, clinical trials have focused on both monotherapy and combination therapy, however poor clinical response to treatment due to acquired resistance have been noted [177,178]. Of these receptors, the interaction between PD-L1 and miRNAs have shown the greatest cause for resistance. p53, via miR-34, which can bind to 3′ UTR of PD-L1, is able to repress PD-L1 [110]. p53 can also regulate miR-200/ZEB1 signaling [179]. Chen et al. found that miR-200/ZEB1 axis regulates PD-L1 expression and has a strong correlation with EMT tumors [180]. Another miRNA that binds and inhibits PD-L1 is miR-140 [111]. miR-140 is downregulated in NSCLC, leading to increased expression of PD-L1, which can increase the expression of cyclin E [111], a gene that dysregulates G1-S transition and the S phase in lung tumors to increase their proliferation [181]. Other reported PD-L1 regulators include miR-197 through the miR-197/CKS1B/STAT3 signaling pathway [182] and an inverse relationship with miR-33a [183]. As for lncRNAs, downregulated HOXA transcript antisense RNA, myeloid-specific 1 (HOTAIRM1) in myeloid-derived suppressor cells (MDSCs) in lung adenocarcinoma decreases the level of homeobox 1 leading to loss of immunosuppressive ability for MDSCs (Figure 1; Table 2) [153]. Altogether, these findings suggest that targeting of specific tumor initiating/suppressive genes by varying non-coding RNAs could have an effect on these immune checkpoints by mimicking the role of immune checkpoint inhibitors. Alternatively, measuring patient response to anti-PD1 therapy in NSCLCs using circRNA and circulating miRNAs has been tested as novel approaches [131,132]. With the ability of circRNA to sponge various miRNA, inducing dysregulation could measure clinical response. As for circulating miRNA, they have the ability to reach various parts of the body and regulate the immune checkpoints, so measuring non-coding RNA expression in patients prior to and during treatment could determine how effective anti-PD1 therapy is. Peng and colleagues reported the strong correlation between immune checkpoints PD1 and CTLA4 with lncRNA MIR155HG, which could potentially serve as models for testing the immune inhibitors prior to clinical trial [184]. Although no other lncRNAs have been shown to have a role in evasion of immune response in lung cancer like HOTAIRM1 and MIR155HG, Denaro et al. created a comprehensive list detailing the role of lncRNAs in other cancers [185]. Thus, it is valuable to study if some lncRNAs play roles in evasion of host immune system in lung cancer.

#### 5.2.5. Role of Non-Coding RNA in Drug Tolerance and Resistance

As previously illustrated, a variety of drugs are available today to treat NSCLC and SCLC, but tumor cells have the unique ability to become drug tolerant and resistant to promote their progression. Activation of EGFR and MET led to upregulation of miR-221/222 and miR-30b/c (Table 1) [100]. Direct repression of apoptotic peptidase activating factor 1 (APAF1) by miR-221/222 and inhibition of BCL2 like 11 (BIM) by miR-30b/c leads to gefitinib resistance in NSCLC [100]. Similarly, MET induced downregulation of miR-103 and miR-203, promotes resistance to EGFR TKI gefitinib via increasing expression of protein kinase C-epsilon, SRC proto-oncogene, non-receptor tyrosine kinase (SRC) and endoribonuclease Dicer [100]. miR-21 upregulation has been found to downregulate PTEN expression during gefitinib treatment, which led to the activation of the phosphoinositide 3-kinase/protein kinase B and mitogen-activated protein kinase/extracellular signal-regulated kinase signaling pathways, known pathways involved in gefitinib resistance in NSCLC tumor cells [101]. Recently, it was reported that upregulated miR-147b in *EGFR* mutant lung adenocarcinoma cells mediates drug tolerance to EGFR TKI osimertinib via repression of *Von Hippel-Lindau* (VHL) and *succinate dehydrogenase* (SDH) [102]. Upregulation of HOXA cluster antisense RNA 3 (HOXA-AS3) in NSCLC confers drug resistance to cisplatin-based chemotherapy via downregulating HOXA3 expression (Table 2) [115]. Additionally, hypoxia-induced miR-155 overexpression leads to enhanced resistance to radiotherapy via downregulating forkhead box O3 (FOXO3A), a tumor suppressor that when unphosphorylated induces apoptosis in tumor cells [103]. In NSCLC tissues, upregulated circFGFR1 expression promotes tumor cells progression and resistance to anti-PD1-based immunotherapy. circFGFR1 directly represses miR-381-3p leading to upregulation of C-X-C motif chemokine receptor 4 (CXCR4) [131,132]. Therefore, non-coding RNA-mediated drug tolerance and resistance need to be investigated in the future.

### 5.3. Role of Non-Coding RNAs and Metabolism in Lung Cancer

**Figure 2:**
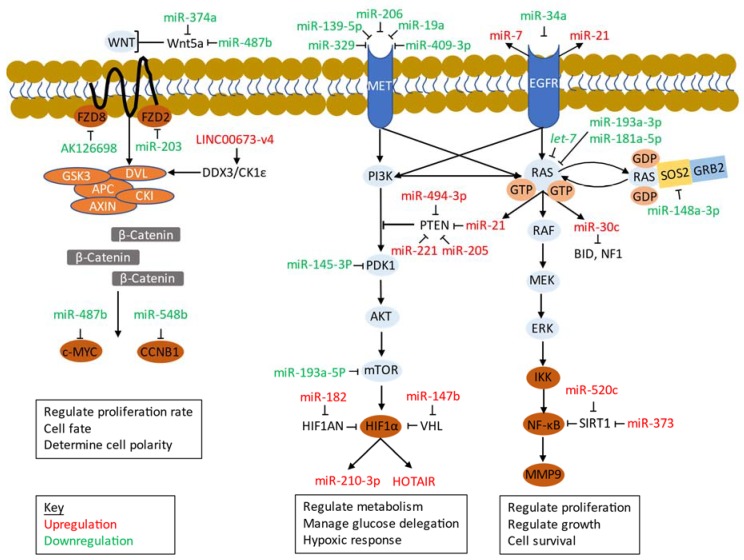
Interactions among cell signaling pathways and non-coding RNAs. →, promotion; ┤, inhibition; ↷/⤾, guanine nucleotide exchange reactions. Abbreviations: AKT serine/threonine kinase (AKT), adenomatous polyposis coli (APC), BH3-interacting domain death agonist (BID), cyclin B1 (CCNB1), casein kinase I (CKI), DEAD-box helicase 3 X-linked (DDX3), disheveled (DVL), epidermal growth factor receptor, frizzled class receptor 2 (FZD2), frizzled class receptor 8 (FZD8), glycogen synthase kinase 3 (GSK3), hypoxia inducible factor 1 subunit alpha (HIF1*α*), hypoxia inducible factor 1 subunit alpha inhibitor (HIF1AN), IκB kinase (IKK), matrix metallopeptidase 9 (MMP9), MET proto-oncogene receptor tyrosine kinase (MET), mechanistic target of rapamycin kinase (mTOR), neurofibromin 1 (NF1), nuclear factor kappa-light-chain-enhancer of activated B (NF-κB), phosphoinositide-dependent protein kinase-1 (PDK1), sirtuin 1 (SIRT1), son of sevenless homolog 2 (SOS2), wingless-type (WNT).

**Figure 3:**
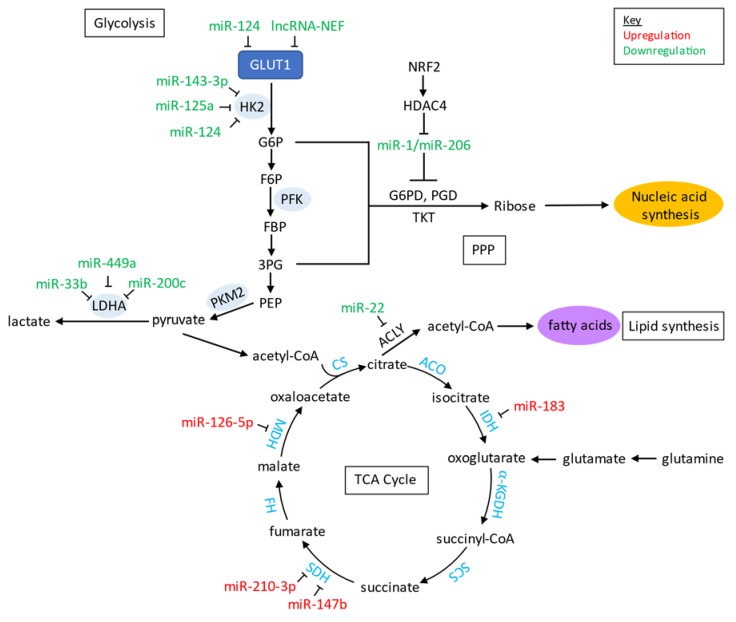
Metabolic pathways including glycolysis, pentose phosphate pathway, tricarboxylic acid cycle, and lipid synthesis with target non-coding RNAs. →, promotion; ┤, inhibition; ⟳, metabolic reactions of the tricarboxylic acid cycle. Abbreviations: 3-Phosphoglyceric acid (3PG), AKT serine/threonine kinase (AKT), ATP citrate lyase (ACLY), aconitase (ACO), ⍺-Ketoglutarate dehydrogenase (⍺-KGDH), citrate synthase (CS), fructose 6-phosphate (F6P), fructose bisphosphatase (FBP), fumarase (FH), glucose 6-phosphate (G6P), glucose-6-phosphate dehydrogenase (G6PD)*,* histone deacetylase 4 (HDAC4), glucose transporter 1 (GLUT1), hexokinase 2 (HK2), isocitrate dehydrogenase (IDH), lactate dehydrogenase A (LDHA), malate dehydrogenase (MDH), nuclear factor erythroid-2-related factor 2 (NRF2), phosphoenolpyruvate (PEP), phosphofructokinase-1 (PFK), phosphogluconate dehydrogenase (PGD), pyruvate kinase M1/2 (PKM2), pentose phosphate pathway (PPP), succinyl-CoA synthetase (SCS), succinate dehydrogenase (SDH), transketolase (TKT), wingless-type (WNT).

For cancer to effectively initiate proliferation and tumorigenicity, various metabolic pathways are altered to support the needs of the cancer cells. When studying the metabolism in lung cancer, there are distinct signaling pathways including apoptotic, growth promoting, and growth inhibiting that must be considered [186]. Out of these pathways the most notable are EGFR, MET, PI3K/Akt/mTOR, Ras/Raf/Mitogen-activated protein kinase/ERK kinase (MEK)/extracellular-signal-regulated kinase (ERK), and Wnt/β-catenin [187] (Table 3; Figure 2). As a result, non-coding RNAs affecting the cell signaling pathways also have a role in regulation of different cell metabolism cycles including glycolysis, pentose phosphate pathway (PPP), tricarboxylic acid cycle (TCA), and lipid synthesis. Targeting of the enzymes within these metabolic cycles by non-coding RNAs has been reported, which could suggest alteration of cellular processes by controlling non-coding RNA regulation. miR-125a and miR-143 have been reported to target the glycolytic enzyme hexokinase 2 (HK2) by downregulation [188]. As for lactate dehydrogenase A, an enzyme in glycolysis responsible for imitation of lactate production, has targets serving as negative regulators include miR-200c [189], miR-33b [190], and miR-449a [191]. Additionally, lncRNA CRYBG3 overexpression has been reported to be associated with LDHA upregulation [192]. Glycolysis harbors another important pathway known as the PPP. Singh et al. looked at regulation of miR-1 and miR-206 by nuclear factor erythroid-2-related factor 2 (NRF2) which serves as a tumor initiator once activated [193]. Once these miRNAs are regulated, progression of the PPP and TCA cycling can occur. As for the TCA cycle, enzymes such as succinate dehydrogenase (SDH), isocitrate dehydrogenase (IDH), and malate dehydrogenase (MDH) have been identified as targets for various miRNAs (Figure 3). Upregulation of miR-147b repressed enzymatic activity of SDH, initiating a pseudohypoxia signaling response [102]. Similarly, upregulation of miR-210 decreased enzymatic activity of SDHD and furthered activity of HIF-1α [194]. This idea of adaptation to stress environments is also present via miR-183 and IDH2 regulation [195]. Vohwinkel et al. compared IDH2 response to elevated CO_2_ with IDH2 response to upregulated miR-183 in epithelial lung cancer cells and found that both downregulated IDH2 [195]. The adaptation of cancer cells to the stress environment by reprogramming metabolic processes could serve as a leading cause of witnessed therapeutic resistance, furthering cancer’s progression. Complexes, such as the miR-182-PDK4 axis, have been reported to regulate pyruvate dehydrogenase which is an essential part of TCA cycling and lipogenesis [196]. Other important targets include miR-22 downregulation on ATP citrate lyase (ACLY), which allowed for ACLY-mediated lipogenesis and caused increased metastatic effects [197]. Enzymatic activity of MDH1 can be altered as well with miR-126-5p in NSCLC, and with greater doses initiated cell toxicity [198]. The role of non-coding RNAs as regulators in a variety of pathways makes them an important part of studying tumor initiation and progression.

#### 5.3.1. EGFR

EGFR is a transmembrane protein. In different types of cancers, the mutation can occur in different spots and for NSCLC it is in the kinase domain [230]. In order to reduce effect of these mutations, therapies focus on targeting with a tyrosine kinase inhibitor (TKI). Due to the position of EGFR on the cell, it serves as an activator site for multiple signaling pathways including MAPK, PI3K/Akt, and PLC-γ1-PKC [230]. Chou and colleagues found that with the overexpression of miR-7, a miRNA induced by EGFR, demonstrated an increase in cell proliferation and tumor growth rate through the Ras/ERK/Myc pathway [231]. EGFR can be directly targeted by miR-34a through upregulation due to the tumor suppressive abilities of miR-34a [199]. EGFR can also dysregulate non-coding RNAs. miR-21 have shown upregulation in NSCLC, demonstrating how EGFR can function as a regulator for potential tumor progressive non-coding RNAs [232]. Alternatively, regulation of crosstalk between pathways has been noted through miR-205 [233]. Migilore et al. investigated MET-TKI resistance and found that with overexpression of miR-205, a target of ERBB receptor feedback inhibitor 1, induced greater EGFR activity [233]. This could suggest the need for co-targeting of EGFR-associated pathways to prevent tumor progression. The targeting of specific non-coding RNAs related to EGFR expression can serve as therapy options in order to inhibit prominent cell signaling pathways present in lung cancer. EGFR has a strong link to glycolysis, which is a precursor to multiple metabolic pathways. Kim et al. looked at how glycolysis was enhanced due to increased glucose uptake and lactate production in order to keep the *EGFR* mutant NSCLC nourished [234]. As a result of high glucose production, ATP levels were increased which suggests glucose fed TCA cycling [234]. By altering metabolism, cancer cells are able to manage themselves and pursue oncogenic processes, so by targeting of specific receptors or enzymes by non-coding RNAs could inhibit these processes and force cancer cells to find alternative resources or simply die.

#### 5.3.2. MET 

The MET signaling pathway can be altered through overexpression of MET and/or its ligand the hepatocyte growth factor, and genetic variation of the MET gene, both common in oncogenic processes [235]. Similar to the EGFR, through the activation of MET there are important downstream pathways including MAPK and PI3K that can be activated [236]. Sun and colleagues looked at miR-329 due to its presence in other cancers, and they found that it targets MET to induce negative regulation, which as a result inhibits proliferation and tumorigenesis of NSCLC [200]. Sun et al. has also noted similar findings with miR-139-5p [201] and miR-206 [202]. Others have found that targeting of c-MET with miR-19a [209] and miR-409-3p [210] could inhibit downstream signaling of the Akt signaling pathway as well. Due to crosstalk between KRAS/MET and EGFR/MET, dual targeting of these signaling pathways via non-coding RNAs could potentially predict drug sensitivity, biomarker potential, and prognostic value [237,238]. 

#### 5.3.3. PI3K/Akt/mTOR

The responsibility of this cell signaling pathway is to regulate metabolism and delegate where glucose should be maintained [239]. Initiation of this pathway is through the activation of membrane receptors including tyrosine kinases (TK) such as EGFR, FGFR, HER2, IGFR-1, PDGFR, and VEGFR [240]. Shi and colleagues found that the lncRNA ROR directly inhibits this pathway and could demonstrate increased sensitivity to cisplatin in NSCLC patients [211]. The role of various lncRNA in regard to lung tumor development and progression is still subject for further study. As for the role of miRNA, it was found that the overexpression of miR-296-3p reduced the level of phosphorylation in this pathway without reducing mRNA expression by targeting apurinic/apyrimidinic endodeoxyribonuclease 1 (APEX1), therefore possibly inhibiting the pathway’s progression particularly in NSCLC [241]. Additionally, miR-296-3p has been reported to have lower levels of expression in comparison to normal lung epithelial cells, and it played a role in inhibiting NSCLC cell proliferation as well as cisplatin sensitivity by targeting C-X3-C motif chemokine receptor 1(CX3CR1) which is upstream of PI3K signaling [242]. miR-142-3p was found to have an association between the PI3K/Akt/mTOR pathway and high mobility group box 1 (HMBG1) induced autophagy, a process of cellular degradation that if in a high presence can demonstrate conflicting results such as promoting tumor survival versus preventing tumorigenesis [212,243]. In this case, NSCLC autophagy was inhibited via the overexpression of miR-142-3p [212]. Another primary target within the PI3k/Akt/mTOR signaling pathway is PTEN. PTEN is a protein found to terminate hyperactive signaling of PI3K, and the loss of its function has been noted in various human cancers [244]. Common non-coding RNA targets of PTEN in lung carcinoma through upregulation include miR-21 [159], miR-205 [245], miR-221 [213], and miR-494 [85]. With targeting of these specific miRNAs, dependent on whether they deactivate or activate PTEN, can work towards understanding the treatments necessary to regulate the PI3K/Akt/mTOR pathway. Non-coding RNAs targeting Akt signaling can also dysregulate cell metabolism. Makinoshima et al. found that there was a link between the PI3K/Akt/mTOR pathway and aerobic glycolysis as well as maintenance of glucose transporter 1 (GLUT1) through optimal membrane localization specifically in *EGFR* mutated lung adenocarcinoma cells [246]. GLUTs are responsible for glucose intake and with increased expression through this pathway can facilitate increased glycolytic activity such as ATP consumption and ACLY stimulation, essentially serving as a precursor for lipid synthesis [247]. Zhao et al. found that with the overexpression of miR-124, GLUT1, and HK2 expression were reduced [214]. Similarly, it was reported that overexpression of lncRNA-NEF reduced expression of GLUT1, resulting in the inhibition of glucose uptake in NSCLC [216]. Targeting GLUTs by non-coding RNAs could potentially have a role in decreasing tumorigenesis. Another contributing factor to metabolism via PI3K/Akt/mTOR signaling is through HIF-1α, mediated by upstream mTOR [248]. Increased HIF-1α can initiate tumorigenesis in lung cancer, but by targeting of VHL by miR-147b [102] and HIF1AN by miR-182 [215], regulation can be reduced to prevent this initiation. HIF-1α can also modulate lncRNA HOTAIR to promote lung tumorigenesis in hypoxic conditions [249,250] and miR-210-3p to prevent HIF-1α degradation via suppression of SDHD enzymatic activity [194]. Focusing on non-coding RNA markers contributory to glucose metabolism and hypoxic response can help better understand how pathway crosstalk influences cell processes leading to tumorigenesis and progression.

#### 5.3.4. Ras/Raf/MEK/ERK (MAPK) 

The MAPK signaling pathway consists of a variety of interconnected pathways that work to regulate growth, proliferation, and survival of the cells, initiated by growth factor receptors, similar to that of the PI3K/Akt/mTOR pathway [251]. Through the inhibition of mTOR/mTORC1, the Ras/Raf/MEK/ERK pathway can be activated through Ras [252]. The MAPK pathway can be activated by decreasing mRNA translation in SIRT1 via miR-520c and miR-373 [253]. The crosstalk between the two pathways perhaps demonstrates how multiple pathways become involved in tumorigenesis and proliferation, and by targeting one can reduce activation of the other. Xie and colleagues looked at miR-148a-3p, a tumor growth suppressor found in NSCLC, and found that it had a role in MAPK/ERK inhibition via overexpression which led to decreased presence of son of sevenless homolog 2 (SOS2) and consequently inhibited Ras activation [203]. Alternatively, targeting activated KRAS with overexpression of tumor suppressors miR-193a-3p [204,205] and miR-181a-5p [206,254] could potentially inhibit further progression of tumor growth. Homogenous *KRAS G12D* mutant, a common mutation causing dysregulation of the MAPK pathway, has been reported to favor glucose fueled TCA cycling due to glucose metabolic reprogramming and reactive oxygen species (ROS) management, leading to increased malignancy [255]. The interplay between miRNA and ROS in cancer treatment response has been discussed in recent reviews [256,257]. Non-coding RNAs such as miR-21 and miR-30c have shown upregulation with KRAS G12D overexpression, which as a result enhance regulation of Ras downstream pathways [207]. In contrast, a negative regulator of Ras includes the *let-7* family which acts as a tumor suppressive miRNA and inhibits downstream signaling [104]. Alternatively, the expression of non-coding RNAs can be regulated through the signaling pathway itself. As noted by Zhang et al., with the inhibition of the MAPK pathways, expression of *Ornlnc1*, a highly expressed lncRNA in *BRAF* mutated cancers, was decreased [208]. As a result, this subsequently reduced cancer cell growth in vivo and in vitro. 

#### 5.3.5. Wnt/β-Catenin 

The Wnt/β-catenin (canonical) pathway holds the responsibility of determining cell polarity, rate of proliferation, and the fate of the cell [258]. Through this pathway, mutation at and surrounding the β-catenin site is most common in cancers, but in regard to lung cancer, its distinguishing factor is based on alterations to various Wnt proteins including Wnt-1–5a, frizzled class receptor 8 (FZD8), and the gene β-catenin [258,259]. miR-487b and miR-203 have been noted to work as tumor suppressive miRNAs in lung cancer by targeting KRAS, WNT5A, SUZ12, MYC, and BMI1 (miR-487b) [218] and FZD2 (miR-203) [219]. Targeting of CCNB1 by miR-548b and Wnt5a by miR-374a have also been reported in lung cancer cell lines, with both serving as tumor repressors [220]. Other lncRNAs that work as a suppressor include MEG3, interaction with p53 to downregulate β-catenin [260], and AK126698, negative regulation of FZD8 [221]. Alternatively, Guan and colleagues found that the overexpression of the lncRNA LINC00673-v4 was found to activate the Wnt/β-catenin pathway, noted by the enhanced interaction between DDX3 and CK1ε essentially leading to enhanced signaling of the pathway in lung adenocarcinoma cells [222]. With the overexpression of β-catenin and β-catenin transcriptional activity by SDH5 (ETC component) inhibition, cancer metabolism can be altered through the mediation of Wnt EMT and metastasis [261]. β-catenin activation is mediated through the GSK-3β enzyme which in turn can be altered through targeting by SDH5 [261]. It has been reported that Wnt/β-catenin can be regulated through the inactivation of the DVL2-NRX complex formation by elevating ROS (Ca^2+^ mediated), which can additionally cause accumulation of nuclear β-catenin in human neural progenitor cells [262,263]. Accumulation of β-catenin through glucose has also been reported to enhance the signaling pathway and as a result increase the risk of cancer development [264]. 

### 5.4. Role of Non-Coding RNAs and Pathogens in Lung Cancer

Due to high exposure rates of pathogens to the lungs, development of NSCLC and SCLC can progress due to resource competition. Conditions such as hepatitis B (HBV), hepatitis C (HCV), Epstein-Barr (EBV), tuberculosis (TB), pneumonia, and other various bacterial/viral infections can lead to such cancer development and progression along with increasing poor clinical outcome. 

Although hepatic infections, specifically hepatitis B and C, have been closely associated with hepatocellular cancers, noted links to lung cancer have been identified. Wu et al. performed a meta-analysis highlighting the problem of acquisition of HBV in lung cancer patients on chemotherapy, which interfered with treatment results [265]. Through targeting of these infections using miRNAs, the risk of lung tumor initiation may be reduced. For HBV, Yao and colleagues found that patients that were being treated with EGFR tyrosine kinase inhibitors developed HBV reactivation during treatment [266]. Non-coding RNAs that target HBV by enhancing infection include overexpression of miR-21 [267], miR-501 [268], lncRNA PCNAP1 [269], and reduced expression of miR-122 [270]. Feng et al. also reported that the expression of miR-154, which is a common tumor suppressor in lung cancer, and PCNAP1 are linked and compete [269]. This suggests that miRNAs and lncRNAs can modulate each other to work towards their advantage. Alternatively, HBV-miR-3 [271] and miR-141 [272] have been found to suppress HBV replication. It has been reported that in HCV associated hepatocellular cancers, there was an upregulation of miR-125a-5p [273]. In contrast, miR-125a-5p has been found to be a tumor suppressor and was downregulated in NSCLC [274]. miR-21, a primary target of PTEN in lung cancers, also demonstrated upregulation in human hepatocytes with HCV [275]. Other key HCV targets include miR-196 [276] and miR-122 [277].

EBV has been clinically found in rare NSCLCs including lymphoepithelioma-like carcinoma (LELC) primarily in east Asian individuals [278,279] as well as reports in lung adenocarcinoma and lung squamous cell carcinoma [280,281]. Unlike cancers, EBV can encode their own non-coding RNAs that have roles in apoptosis, gene regulation, cell signaling, host-cell immunity, and cancer cell proliferation [282]. These include EB virus-encoded RNAs (EBERs), BamHI-A rightward transcripts (BARTs), viral snoRNA1, EBV-sisRNA-1, and EBV-miRNAs [283]. Movassagh and colleagues reported that out of the tested lung adenocarcinoma and lung squamous cell carcinoma samples the expression of EBV miRNA was 6% and 12% respectively [284]. Similarly, high levels of EBV miRNA have not been detected in NSCLC, suggesting that a source of prior infection of EBV in lung cancer patients is the cause for the presence and there is no current associated link between infection and tumorigenesis [285]. The role of EBV noncoding RNAs on varying lung cancers is subject to further study.

The role of pulmonary diseases including pneumonia and TB on lung cancer development have been studied to improve clinical results. However, the coexistence between lung cancer and pulmonary TB have shown conflicting results. These results can be split into categories such as 1. TB initiating lung cancer development, 2. Reactivation of TB in lung cancer patients and 3. Radiological and physiological similarities between TB and lung cancer [286]. Abd-El-Fattah et al. tested expression of miRNA in serum of lung cancer patients to determine possible association with pulmonary diseases [287]. They found that upregulation of miR-21 and miR-155, both having a role in inflammation rate, could serve as a source of TB and pneumonia, and adjacently high expression levels in patients with pneumonia [287]. Furthermore, testing of the effect of potential pneumonia and TB non-coding RNA biomarkers in comparison to non-coding RNA expression in lung cancers could serve as beneficial for determining future treatment plans.

## 6. Cell-Free Circulating Non-Coding RNAs in Lung Cancer

Circulating non-coding RNAs have been identified as valuable noninvasive biomarkers for early detection and analyzing clinical response due to their presence in human body fluids such as serum, urine, and plasma. Prognostic value and biomarker potential in lung cancer patients have been studied by altering expression levels and measuring established expression levels of miR-21 [288], miR-145 [289], miR-125b [290], miR-182 [291], and miR-19b [292]. In correspondence to drug response, low expression of miR-145 was found to be associated with chemotherapy resistance [289] while downregulation of miR-21 demonstrated better gefitinib response [288]. For long non-coding RNAs, HOTAIR [293], LINC00152 [294], growth arrest-specific transcript 5 (GAS5) [295,296], lncRNA-NEF [216], and SOX2 overlapping transcript (SOX2OT) [296] have been identified as potential biomarkers for lung cancers. With further research on circulating non-coding RNAs in relation to particular cancers, there could possibly be greater early detection using this noninvasive technique. This could account for starting treatments earlier, prolonging length of survival, and monitoring the effectiveness of treatment based on RNA regulation.

## 7. Pre-Clinical Models for Human Lung Cancer

In order to test treatments prior to clinical study, researchers have developed in vivo and in vitro pre-clinical models such as spheroid cultures, organoid cultures, patient derived explants/xenografts, and air-liquid interface models. Spheroid cultures serve as 3-D models that can demonstrate cell aggregation and tumor progression. Kim et al. looked at the role of miR-34 on cancer cell invasion by using spheroid cultures and found that miR-34b/c significantly suppressed spheroid invasion and EMT [297]. Ekert and colleagues compared 3-D spheroid cultures with 2-D monolayer cultures for multiple *EGFR* wild type and mutated cell lines and found that 3-D spheroids were able to reproduce proliferative processes. However the spheroids had higher basal receptor phosphorylation activity of EGFR and c-MET, and altered therapeutic response in multiple cell lines compared to the 2-D models [298]. Differences in drug response in 3-D models compared to 2-D models has been reported in multiple studies, so properly accounting for these alterations will need to be further evaluated prior to moving towards clinical trial. Alternatively, organoid cultures can also be used as 3-D models. Kim et al. compared 3-D organoids with patient derived models and found that organoids supported differing histology and genetic characteristics of various lung cancer types, however drug response continued to vary in these models similar to spheroids [299]. Similarly, Sachs et al. reported that organoid cultures can maintain cancer-related gene mutations, both apoptotic and proliferating [300]. Due to limited current data, future study of targeting common cancer initiating genes with non-coding RNAs using organoid models could serve as promising indicators for clinical therapeutic response and tumorigenesis. A form of in vitro modeling includes air-liquid interface models, which attempt to mimic the microenvironment of the lungs [301]. Movia et al. utilized these multilayered cultures to test response chemotherapy administered through inhalation and they found that these models demonstrated high chemoresistance [301]. Further study of therapeutic efficacy using miRNAs by mimicking the lung microenvironment with air-liquid interface models could serve as promising future study. 

## 8. Clinical Trial of Lung Cancer

Although there have been many reported clinical trials involving non-coding RNAs as early detection biomarkers and diagnostic indicators, there are few involving non-coding RNAs and treatment [302]. The first approved miRNA target therapy was MRX34, which is a miR-34a mimic, utilized on hepatocellular carcinoma, lung cancer and others with hepatic metastasis (NCT01829971) [303]. With this clinical trial, feasibility of non-coding RNAs as potential therapies was confirmed. In a trial from The Asbestos Diseases Research Institute (NCT02369198), looked at the linkage of miRNA expression with malignant pleural mesothelioma, a rare type of lung cancer [304]. Using this study, they test miRNAs from the miR-15 family and drug sensitivity to determine whether treatment with altering regulation of non-coding RNAs would be beneficial for long term treatment. To accomplish this, they utilized synthetic miRNA known as TargomiRs, specifically a miR-16 mimic. Out of the 26 individuals who participated, only one demonstrated a partial response to the treatment. In another clinical study by Berghmans et al., patients with NSCLC were tested with combination chemotherapy treatment Cisplatin and Vinorelbine [305]. They attempted to determine if mRNA and miRNA could be determinant biomarkers for prognostic value. Comparing transcriptomic analyses from previous study of potential miRNA biomarkers [306] versus actual expression in the patients did not demonstrate clear results. With limitations due to varying histology of lung cancers and isolation of miRNA expression, determining clear biomarkers is difficult for researchers to identify. As progression of therapy using non-coding RNAs in 3-D models persist, there will be anticipation for more clinical trials. At this current stage, involvement of non-coding RNAs as promising therapies is unknown. 

## 9. Concluding Remarks and Future Direction

Through our analysis of research regarding non-coding RNAs we found that there is indeed a link between miRNAs and lncRNAs with lung tumor initiation and progression. However, further research is needed, especially in regard to lncRNAs. It is imperative that lncRNAs are studied to further our understanding of their mechanisms of action within lung cancer, especially since lncRNAs and miRNAs have been found to affect each other and lung cancer overall, as exemplified by RNA component of mitochondrial RNA processing endoribonuclease being able to affect miR-206 levels to activate oncogenes in lung cancer (see Table 2). As shown before, literature reviews such as “Long noncoding RNAs as regulators of cancer immunity” have summarized the role of non-coding RNAs in other types of cancers, and researchers can use this information to determine whether those non-coding RNAs are also able to affect lung cancer [89,163,185]. Furthermore, new and emerging technologies such as RNA editing and RNA modification warrant further research as they too have been implicated in altering lncRNAs and miRNAs to either promote or impede lung tumor initiation and progression. Altogether, the aforementioned challenges must be addressed in order to decrease the perverse effects of lung cancer, especially since traditional therapeutic methods employed to obstruct lung cancer, such as chemotherapy and targeted therapy (e.g., EGFR TKIs) have in part been made obsolete by the expression of non-coding RNAs. Due to emerging understanding of cell signaling/metabolic mechanisms, catering target therapy to noninvasive methods such as altering non-coding RNA expression or indicating specific biomarkers (e.g., circulating non-coding RNAs) could serve as new effective methods [307]. While most of the past clinical studies focused on non-coding RNAs as diagnostic markers, new emerging clinical trials are moving towards understanding how non-coding RNAs can be used as prognostic and clinical response markers [308]. Using computer science-based artificial intelligence [309], models can be created to predict dysregulation in different microenvironments. Limitations include coming up with a standardized collection of specific biomarkers due to differing histology in various cancer types, but using artificial intelligence could potentially source various databases and isolate the most effective non-coding RNAs. Not only is there a challenge with differing histology and microenvironments, but non-coding RNAs can be regulated differently between immune cells, tumor cells, and cells from other cancers. A potential way to combat this is by utilizing crosstalk between lncRNA/miRNA, miRNA/miRNA, or lncRNA/lncRNA modulation, similar to combination therapy. With more advanced clinical models that mimic the lung tumor microenvironment, the value non-coding RNAs and therapeutic treatment can be monitored and hopefully move towards more clinical trials. 

## Figures and Tables

**Table 1:** miRNAs involved in lung cancer initiation and progression.

miRNA	Lung Cancer Subtype	Downstream Direct Targets	Hallmark	Initiation (I)/Progression (P)	References
Upregulated miRNAs					
miR-1246, miR-1290	LUAD	MT1G	Enrichment of TICs	I and P	[83]
miR-21	NSCLC	PDCD4, APAF1	Inactivation of Tumor Suppressor	I	[90]
miR-31	NSCLC	RASA1, SPRED1/2, SPRY1/3/4	Inactivation of Tumor Suppressor	I	[91]
miR-17~92	SCLC	PTEN, RBL2	Inactivation of Tumor Suppressor	I	[92]
miR-196a	NSCLC	HOXA5	Promotion of Metastasis	P	[93]
miR-18a-5p	NSCLC	IRF2	Promotion of Proliferation and Inhibition of Apoptosis	I	[94]
miR-25, miR-93, miR-106b	NSCLC	p21	Promotion of Proliferation	P	[95]
miR-19a	NSCLC	PTEN	Promotion of EMT and Metastasis	P	[96]
miR-130b	NSCLC	TIMP2	Promotion of Metastasis	P	[97]
miR-574-5p	SCLC	PTPRU	Promotion of Metastasis	P	[98]
miR-494	NSCLC	PTEN	Promotion of Angiogenesis	P	[99]
miR-221/222	NSCLC	APAF1	Promotion of Drug Resistance to EGFR TKI gefitinib	P	[100]
miR-30b/c	NSCLC	BIM	Promotion of Drug Resistance to EGFR TKI gefitinib	P	[100]
miR-21	NSCLC	PTEN	Promotion of Drug Resistance to EGFR TKI gefitinib	P	[101]
miR-147b	LUAD	VHL, SDH	Promotion of Drug Tolerance to EGFR TKI osimertinib	P	[102]
miR-155	NSCLC	FOXO3A	Promotion of Resistance to Radiotherapy	I	[103]
Downregulated miRNAs					
miR-184	SCLC	EPAS1/HIF-2α	Promotion of Metastasis	P	[98]
miR-103, miR-203	NSCLC	PKC-ε, SRC and Dicer	Promotion of Drug Resistance to EGFR TKI gefitinib	P	[100]
*let-7*	NSCLC	RAS	Activation of Oncogene	I	[104]
miR-33	NSCLC	METTL3	Promotion of RNA Modifications	I and P	[105]
miR-193a-5p	NSCLC	PIK3R3, mTOR	Promotion of Metastasis	P	[106]
miR-193a-3p	NSCLC	ERBB4, S6K2	Promotion of Metastasis	P	[106]
miR-335	SCLC	RANKL	Promotion of Metastasis	P	[107]
miR-126	LUAD	VEGFA	Promotion of Angiogenesis	P	[108]
miR-195	LUSC	VEGFA	Promotion of Angiogenesis	P	[109]
miR-34a/b/c	NSCLC	PDL1	Evasion of Host Immune Response	P	[110]
miR-140	NSCLC	PDL1, CCNE1	Evasion of Host Immune Response	P	[111]

Abbreviations: Apoptosis peptidase activating factor 1 (APAF1), BCL-2-like protein 11 (BIM), cyclin E1 (CCNE1), endoribonuclease dicer (DICER), endothelial PAS domain protein 1 (EPAS1), Erb-B2 receptor tyrosine kinase 4 (ERBB4), hypoxia-inducible factor 1 α (HIF-1α), homeobox A5 (HOX5A), interferon regulatory factor 2 (IRF2), forkhead box O3 (FOXO3), lung adenocarcinoma (LUAD), lung squamous cell carcinoma (LUSC), methyltransferase like 3 (METTL3), mechanistic target of rapamycin kinase (mTOR), metallothionein 1G (MT1G), MYC proto-oncogene (MYC), non-small cell lung carcinoma (NSCLC), phosphatase and tensin homolog (PTEN), programmed cell death 4 (PDCD4), phosphoinositide-3-kinase regulatory subunit 3 (PIK3R3), protein kinase C epsilon (PKC-ε), programmed cell death 1 ligand 1 (PDL1/CD274), protein tyrosine phosphatase receptor type U (PTPRU), tumor necrosis factor ligand superfamily member 11 (RANKL), RAS p21 protein activator 1 (RASA1), RB transcriptional corepressor like 2 (RBL2), sprouty related EVH1 domain containing 1 (SPRED1), sprouty 1 (SPRY1), SRC proto-oncogene non-receptor tyrosine kinase (SRC), TIMP metallopeptidase inhibitor (TIMP2), small cell-lung cancer (SCLC), vascular endothelial growth factor A (VEGFA), ribosomal protein S6 kinase B2 (S6K2), von Hipper-Lindau tumor suppressor (VHL).

**Table 2:** Long non-coding RNAs and circular RNAs involved in lung cancer initiation and progression.

LncRNA and circRNA	Lung Cancer Subtype	Downstream Direct Targets	Hallmark	Initiation (I)/Progression (P)	References
Upregulated LncRNAs					
HOTAIR	NSCLC	N.A.	Enrichment of TICs	I	[88]
SBF2-AS1	NSCLC/SCLC	SUZ12, EZH2	Promotion of Tumor Cell Proliferation	P	[112]
LINC00312	LUAD	YBX1	Promotion of Vasculogenic Mimicry and Metastasis	P	[113]
MEG3	NSCLC	JARID2	Promotion of TGFβ-induced EMT	I and P	[114]
HOXA-AS3	LUAD, LUSC	HOXA3	Promotion of Drug Resistance to Platinum-based Chemotherapy	P	[115]
SOX2OT	LUSC	N.A.	Promotion of Tumor Cell Proliferation	P	[116]
H19	NSCLC	miR-107	Promotion of Tumor Cell Proliferation	P	[117]
ANRIL	NSCLC	EZH2	Promotion of Tumor Cell Proliferation and Metastasis	P	[118]
CAR10	LUAD	miR-30, miR-203	Promotion of Metastasis	P	[119]
MALAT1	NSCLC	miR-204	Promotion of Metastasis	P	[120]
PVT1	NSCLC	miR-195	Promotion of Resistance to Radiotherapy	P	[121]
BC087858	NSCLC	N.A.	Promotion of Drug Resistance to EGFR TKI gefitinib	P	[122]
Downregulated LncRNAs					
LINC01186	NSCLC	N.A.	Inhibition of TGFβ-induced EMT	P	[123,124]
LincRNA-p21	LUAD	N.A.	Promotion of Hypoxia-induced Angiogenesis	P	[125]
HOTAIRM1	LUAD	HOXA1	Inhibition of Evasion of Hosts Immune Response	P	[126]
PANDAR	NSCLC	NF-YA	Inhibition of Tumor Cell Proliferation	P	[127]
MIR22HG	NSCLC	YBX1	Inhibition of Tumor Cell Proliferation	P	[128]
GAS5	NSCLC	N.A.	Inhibition of Tumor Cell Proliferation, Activation of Tumor Suppressors	I/P	[129]
GAS5	NSCLC	IGF-1R	Promotion of Apoptosis, Inhibition of Drug Resistance to EGFR TKI gefitinib	P	[130]
Upregulated circRNAs					
circFGFR1	NSCLC	miR-381-3p	Promotion of Progression and Resistance to Immune Checkpoint Inhibitors	P	[131,132]
circ_0020123	NSCLC	miR-144	Promotion of Tumor Cell Proliferation	P	[133]
circTP63	LUSC	miR-873-3p	Promotion of Tumor Cell Proliferation	P	[134]
circPRKCI	LUAD	miR-545, miR-589	Promotion of Tumor Cell Proliferation, Promotion of Drug Resistance to EGFR TKI gefitinib	P	[135]
circ_0000064	NSCLC	N.A.	Promotion of Tumor Cell Proliferation and Metastasis, Inhibition of Cell Apoptosis	P	[136]
F-circEA	NSCLC	N.A.	Promotion of Metastasis	P	[137]
circ_0067934	NSCLC	N.A.	Promotion of EMT and Metastasis	P	[138]
CDR1as	NSCLC	miR-7	Activation of Oncogene, Inhibition of Apoptosis	P	[139]
circ_103809	NSCLC	miR-4302	Activation of Oncogene	P	[140]
Downregulated circRNAs					
cir-ITCH	NSCLC	miR-7, miR-214	Inhibition of Tumor Cell Proliferation	P	[141]
circ_100395	NSCLC	miR-1228	Inhibition of Tumor Cell Proliferation and Metastasis	P	[142]
circ_0033155	NSCLC	PTEN	Inhibition of Tumor Cell Proliferation and Metastasis	P	[143]
circRNF13	NSCLC	miR-93-5p	Inhibition of Metastasis	P	[144]

Abbreviations: antisense non-coding RNA in the INK4 locus (ANRIL), BCL-2-like protein 4 (BAX), chromatin-associated RNA 10 (CAR10), enhance of zeste 2 polycomb repressive complex 2 subunit (EZH2), growth arrest-specific transcript 5 (GAS5), homeobox A1 (HOXA1), HOX transcript antisense RNA (HOTAIR), HOXA transcript antisense RNA, myeloid-specific 1 (HOTAIRM1), HOXA cluster antisense RNA 3 (HOXA-AS3), insulin like growth factor receptor 1 (IGF-1R), homeobox A3 (HOXA3), Jumonji and at-rich interaction domain containing 2 (JARID2), lung squamous cell carcinoma (LUSC), lung adenocarcinoma (LUAD), metastasis-associated lung adenocarcinoma transcript 1 (MALAT1), maternally expressed 3 (MEG3), nuclear transcription factor Y, alpha (NF-YA), non-small cell lung carcinoma (NSCLC), promoter of CDKN1 antisense DNA damage-activated RNA (PANDAR), phosphatase and tensin homolog (PTEN), plasmacytoma variant translocation 1 (PVT1), small cell lung carcinoma (SCLC), SOX2 overlapping transcript (SOX2OT), SUZ12 polycomb repressive complex 2 subunit (SUZ12), Y-Box binding protein 1 (YBX1), not available, (N.A.).

**Table 3:** Non-coding RNAs that affect cell signaling and metabolic pathways.

Name of ncRNA	Type of ncRNA	Targets	Signaling Pathway	Metabolic Pathway	References
miR-21	miRNA	PTEN	Ras/Raf/MEK/ERK, PI3K/Akt/mTOR	Glycolysis, Glucose-mediated TCA Cycle	[101]
miR-147b	miRNA	VHL, SDH	EGFR	ETC, TCA Cycle	[102]
miR-34a	miRNA	EGFR	Ras/Raf/MEK/ERK, PI3K/Akt/mTOR	Glycolysis	[199]
miR-329	miRNA	MET	Ras/Raf/MEK/ERK, PI3K/Akt/mTOR	N.A.	[200]
miR-139-5p	miRNA	MET	Ras/Raf/MEK/ERK, PI3K/Akt/mTOR	N.A.	[201]
miR-206	miRNA	MET	Ras/Raf/MEK/ERK, PI3K/Akt/mTOR	N.A.	[202]
miR-148a-3p	miRNA	SOS2	Ras/Raf/MEK/ERK	N.A.	[203]
miR-193a-3p	miRNA	KRAS	Ras/Raf/MEK/ERK	N.A.	[204,205]
miR-181a-5p	miRNA	KRAS	Ras/Raf/MEK/ERK	N.A.	[206]
miR-30c	miRNA	BID, NF1	Ras/Raf/MEK/ERK	Glucose-mediated TCA Cycle	[207]
*Orilnc1*	lncRNA	N.A.	Ras/Raf/MEK/ERK	N.A.	[208]
miR-494-3p	miRNA	PTEN	PI3K/Akt/mTOR	Glycolysis	[85]
miR-19a	miRNA	MET	PI3K/Akt/mTOR	N.A.	[209]
miR-409-3p	miRNA	MET	PI3K/Akt/mTOR	N.A.	[210]
ROR	lncRNA	N.A.	PI3K/Akt/mTOR	Glycolysis	[211]
miR-142-3p	miRNA	HMGB1	PI3K/Akt/mTOR	Glycolysis	[212]
miR-221	miRNA	PTEN	PI3K/Akt/mTOR	Glycolysis	[213]
miR-124	miRNA	GLUT1, HK2	PI3K/Akt/mTOR	Glycolysis	[214]
miR-182	miRNA	HIF1AN	PI3K/Akt/mTOR	ETC, TCA Cycle	[215]
lncRNA-NEF	lncRNA	GLUT1	PI3K/Akt/mTOR	Glycolysis	[216]
miR-145-3p	miRNA	PDK1	PI3K/Akt/mTOR	N.A.	[217]
miR-487b	miRNA	KRAS, Wnt5a, MYC	Wnt/β-Catenin	N.A.	[218]
miR-203	miRNA	FZD2	Wnt/β-Catenin	N.A.	[219]
miR-548b	miRNA	CCNB1	Wnt/β-Catenin	N.A.	[220]
miR-374a	miRNA	Wnt5a	Wnt/β-Catenin	N.A.	[220]
AK126698	lncRNA	FZD8	Wnt/β-Catenin	ETC	[221]
LINC00673-v4	lncRNA	DDX3	Wnt/β-Catenin	N.A.	[222]
miR-660	miRNA	MDM2	p53	N.A.	[223]
miR-98miR-453	miRNA	TP53	p53	N.A.	[224]
circ-MTO1	circRNA	miR-17 ┤QKI-5	Notch	N.A.	[225]
circRNA_103809	circRNA	miR-4302 ┤ZN121	MYC	N.A.	[140]
miR-342-3p	miRNA	E2F1	MYC	N.A.	[226]
miR-451a	miRNA	MYC	MYC	N.A.	[227]
PART1	lncRNA	miR-635	JAK/STAT	N.A.	[228]
miR-135	miRNA	TRIM16	JAK/STAT	N.A.	[229]

┤, inhibition. Abbreviations: AKT serine/threonine kinase (AKT), BH3-interacting domain death agonist (BID), C-X3-C motif chemokine receptor 1 (CX3CR1), DEAD-box helicase 3 X-linked (DDX3), epidermal growth factor receptor (EGFR), E2F transcription factor 1 (E2F1), electron transport chain (ETC), frizzled class receptor 2 (FZD2), frizzled class receptor 8 (FZD8), glucose transporter 1 (GLUT1), hypoxia inducible factor 1 subunit alpha inhibitor (HIF1AN), high mobility group box 1 (HMGB1), janus kinase (JAK), mouse double minute 2 (MDM2), mechanistic target of rapamycin kinase (mTOR), neurofibromin 1 (NF1), prostate androgen-regulated transcript 1 (PART1), phosphoinositide-dependent protein kinase-1 (PDK1), phosphatase and tensin homolog (PTEN), quaking homolog (QKI-5), sirtuin 1 (SIRT1), son of sevenless homolog 2 (SOS2), signal transducer and activator of transcription (STAT), tricarboxylic acid cycle (TCA Cycle), tumor protein p53 (TP53), Wnt family member 5a (Wnt5a), wingless-type (WNT), not available (N.A.).
